# News and views

**DOI:** 10.1111/iwj.14068

**Published:** 2022-12-28

## 1 3M

### New 3M Ambulatory Potentially Preventable Complications software supports growing need for patient safety and quality oversight in outpatient settings

3M Health Information Systems (HIS) announced the rollout of its new methodology, 3M™ Ambulatory Potentially Preventable Complications (3M AM‐PPCs) software, developed to address patient safety and quality oversight for procedures performed in hospital outpatient departments or ambulatory surgery centers.

Unlike the inpatient setting, the outpatient environment has a less mature patient safety measurement system. As the scope, volume, and complexity of procedures conducted in ambulatory settings expand, the need for a comprehensive approach to identify and trend adverse events also increases. The new 3M AM‐PPCs software uses sophisticated grouping logic and allows providers and payers to identify and analyse complications in outpatient settings by specific procedures, service lines, providers, and facilities. National benchmarks are also included. These robust drill‐down data analysis capabilities can provide insights on patient safety areas with the greatest opportunities for quality and excess cost improvements in outpatient settings.

“3M AM‐PPCs can help payers and providers develop interventions to reduce preventable complications, improve care in ambulatory settings, and establish targeted incentives to guide outcomes‐based payment,” said Dr. Sandeep Wadhwa, 3M HIS global chief medical officer. “This new software can also facilitate easy and accurate reporting of ambulatory complications for public report cards and quality improvement efforts by providing a fair, age‐adjusted basis for comparing service lines, hospital outpatient procedures and ambulatory surgery centers.”

Complications not only impact the quality of care, but also directly impact resource utilisation and contribute to potentially avoidable health care costs. They are associated with excess follow‐up visits, emergency department visits, and inpatient admissions and can affect patient safety, satisfaction, and emotional health. Although not all complications are preventable, excessive complications can be reduced if they are clearly identified and addressed. Likewise, the solution also identifies providers and sites of excellence with lower‐than‐expected complications, from whom best practises and recognition can follow.

“Patient safety is a priority for all health care organizations. As the trend toward ambulatory care delivery continues to grow, the need to promote safety and efficiency in this setting will also continue to grow. Our preliminary analysis of more than 11 million at‐risk ambulatory procedures indicates complication rates may be as high as 10 percent for certain procedures, with significant variation by hospital outpatient departments, which suggests there is room for improvement in the quality of care being delivered,” said Dr. Wadhwa.

## 2 JOHNSON & JOHNSON

### Innovativeness. Social Responsibility. Learn how these and other key attributes landed the company on the global corporate reputation list for the 20th year in a row and ranked the company #1 on the Pharmaceutical Industry list for the ninth consecutive year

For more than 135 years, Johnson & Johnson has been dedicated to addressing some of the world's toughest health challenges. And this unwavering commitment has not gone unnoticed: Fortune just ranked Johnson & Johnson as a Top 50 All‐Star on its World's Most Admired Companies list for the 20th consecutive year. For 2022, the company ranked #1 on the Pharmaceutical Industry list for the ninth year in a row and in the top 20 on the overall Top 50 All‐Stars list.

Guided by their Credo, Johnson & Johnson's mission statement, the company's enduring goal has been to change the trajectory of human health. And Johnson & Johnson has been relentlessly driving toward that goal through such efforts as its ground‐breaking work to help eliminate cancer, its support of health equity solutions, and its pandemic response.

In 2021, Johnson & Johnson's COVID‐19 vaccine received Emergency Use Authorization from the U.S. Food and Drug Administration. That same year, the Janssen Pharmaceutical Companies of Johnson & Johnson also had two new product approvals, including one for relapsing forms of multiple sclerosis. The other, for non‐small cell lung cancer in patients with a specific genetic mutation, is just one example of how the company is making progress toward its vision of a world without cancer.

Last year, the company also kicked off its Health Equity Innovation Challenge to find and fast‐track advances that have the potential to diminish healthcare disparities in the U.S. The Challenge follows the launch of Our Race to Health Equity, through which Johnson & Johnson announced in late 2020 that it was committing $100 million over the next 5 years to invest in and promote health equity solutions for black people and other people of colour in the U.S.

Johnson & Johnson has always aimed to put a world of well within reach of everyone, everywhere. It is honoured to be named one of Fortune's World's Most Admired Companies once again.

## 3 RMIT UNIVERSITY AND BOLTON CLARKE RESEARCH INSTITUTE

### A thermal‐imaging tool to screen for chronic wounds could enable nurses to identify these hard‐to‐heal sores during the first assessment at a person's home

Nearly half a million Australians live with chronic wounds, which greatly affect their quality of life and cost the nation's health system around $3 billion each year. The latest innovation by researchers at RMIT University and Bolton Clarke Research Institute builds on their team's work published last year, which enabled the identification of chronic leg sores by the second week after the baseline assessment.

Their latest published results allow the identification of these wounds a week earlier and represent a significant leap forward, the team says. Lead researcher Professor Dinesh Kumar said their latest clinical study, published in the Nature journal *Scientific Reports*, presents an AI‐powered system to predict how leg ulcers will heal based on thermal images from the first assessment. Co‐researcher, RMIT's Dr Quoc Cuong Ngo, said while thermal imaging had previously been considered for detecting chronic wounds, the team's methods enabled significantly earlier detection than other approaches that have been researched.

The new method provides information on the spatial heat distribution in a wound and predicts, with 78% accuracy, whether leg ulcers will heal in 12 weeks without specialised treatment.

Wounds change significantly over the healing trajectory—higher temperatures signal potential inflammation or infection, while lower temperatures can indicate a slower healing rate due to decreased oxygen in the region. The research was based on thermal images collected from 56 clients with venous leg ulcers, a type of ulcer associated with poor vein function. This type of ulcer is the most common chronic wound in Australia.

The current gold‐standard approach requires taking tracings of the wound size after 4 weeks, involving physical contact with the wound, which delays the identification of slow‐healing wounds.

Bolton Clarke Research Institute Senior Research Fellow Dr Rajna Ogrin said the non‐contact method reduces infection risk by minimising physical contact. “Clinical care is provided in many different locations, including specialist clinics, general practices and in people's homes,” she said. “This method provides a quick, objective, non‐invasive way to determine the wound‐healing potential of chronic leg wounds that can be used by healthcare providers, irrespective of the setting. “This means specialised treatments, including advanced wound‐cleaning techniques and therapies, can be implemented immediately for problematic leg wounds—up to 4 weeks earlier than the current gold standard.”

